# In Vitro Characterization of *Centella asiatica* Extracellular Vesicles and Their Skin Repair Effects in a UVB-Irradiated Mouse Model

**DOI:** 10.3390/ijms26188982

**Published:** 2025-09-15

**Authors:** Tsong-Min Chang, Chung-Chin Wu, Huey-Chun Huang, Shr-Shiuan Wang, Ching-Hua Chuang, Pei-Lun Kao, Wei-Hsuan Tang, Luke Tzu-Chi Liu, Wei-Yin Qiu, Ivona Percec, Charles Chen, Tsun-Yung Kuo

**Affiliations:** 1 Department of Applied Cosmetology, HungKuang University, Taichung City 433304, Taiwan; 2 Schweitzer Biotech Company, Taipei City 114066, Taiwan; 3 Department of Medical Laboratory Science and Biotechnology, China Medical University, Taichung City 406040, Taiwan; 4 Department of Clinical Application, Center for iPS Cell Research and Application (CiRA), Kyoto University, Kyoto 606-8507, Japan; 5 Division of Plastic Surgery, Department of Surgery, University of Pennsylvania, Philadelphia, PA 19010, USA; 6 College of Science and Technology, Temple University, Philadelphia, PA 19122, USA

**Keywords:** *Centella asiatica*, skin care, extracellular vesicles, antioxidants, cosmetics, anti-inflammatory, skin whitening, UV damage, photoaging

## Abstract

This study characterized extracellular vesicles (EVs) isolated from medicinal herb *Centella asiatica* tissue culture and investigated their therapeutic properties using in vitro assays and a ultraviolet (UV)-induced damage mouse model. EVs were isolated from *C. asiatica* tissue culture and characterized by nanoparticle tracking analysis, and cytotoxicity, antioxidant, anti-melanin, and anti-inflammation properties were evaluated by in vitro assays. *C. asiatica* EVs were found to contain high levels of polyphenols and mitigate hydrogen peroxide-induced intracellular reactive oxygen species (ROS). The EVs were further able to reduce intracellular melanin content and tyrosinase activity. They exhibited anti-inflammatory effects by downregulating the expression of pro-inflammatory genes, COX2, as well as nitric oxide production. In the UV-induced photodamage mouse model, gels with or without EVs were applied to the UV-damaged site, skin appearance was observed daily, and skin histopathology was analyzed on day 7. In mice with UV-induced skin damage, the daily application of *C. asiatica* EV gel reduced skin epidermis thickness and inflammation compared to UV-only or blank gel at seven days after UV irradiation. The beneficial effects of *C. asiatica* EVs on skin quality warrant further studies as promising agents in skin care applications.

## 1. Introduction

*Centella asiatica*, also commonly known as Gotu Kola, is a traditional medicinal herb widely used across Asia for promoting wound healing, reducing inflammation, and improving skin appearance [1]. Its properties are primarily attributed to phytochemicals, including triterpenoids, flavonoids, and polyphenols, which have been reported to modulate collagen synthesis, melanogenesis, and oxidative stress responses in vitro and in animal models [2,3,4]. In modern dermatology and cosmetics, *C. asiatica* extracts are incorporated into formulations targeting photoaging, hyperpigmentation, and skin barrier repair [4,5]. However, crude plant extracts are often compounded by variability due to environmental, seasonal, and processing factors, which can affect reproducibility and efficacy.

Extracellular vesicles (EVs) are nanoscale lipid bilayer membrane-bound particles secreted by cells into the extracellular environment, carrying bioactive proteins, nucleic acids, lipids, and secondary metabolites. They can modulate physiological and pathological processes of target cells [6]. EVs have emerged as novel bioactive delivery systems, offering enhanced stability, cellular uptake, and potential for targeted action compared to naked drugs or crude extracts [7,8]. Plant-derived EVs (PDEVs) are of considerable interest as an alternative to mammalian EVs due to their comparable advantages in safety, scalability, cost-effectiveness, and ethical sourcing [9,10]. PDEVs are produced in plant cells in response to fungal infection, cell-to-cell communication, and cell wall remodeling and originate from the endosomal system (multivesicular body) and plasma membrane of plant cells, similar to the biogenesis pathways of mammalian EVs [11]. However, the presence of a cell wall necessitates plant-specific mechanisms such as exocyst-positive organelles and vacuolar pathways to traffic vesicles through plasmodesmata, apoplast, or remodeling of the cell wall to facilitate their release [10,11].

Plant-derived RNA, particularly microRNAs (miRNAs), can be found in EVs and play a significant role by modulating gene expression in recipient cells [11]. Despite the growing interest in plant EVs, research on *C. asiatica* EVs remains limited, and their potential roles in skin health are not yet fully characterized. It was only recently that exosome-like EVs from plants, including *C. asiatica*, were shown to promote skin improvement and wound healing more effectively than conventional plant extracts [12,13].

Plant tissue culture provides a sustainable and reproducible source of biomass under controlled conditions, eliminating seasonal and environmental variability and enabling standardized EV production at scale [14]. However, the comprehensive in vitro and preclinical characterization of tissue culture–derived *C. asiatica* EVs for cosmetics remains limited. This study aims to fill this gap by investigating the in vitro properties of EVs isolated from *C. asiatica* tissue culture as well as the effects of *C. asiatica* EV-based gel on UV-induced skin damage in a mouse model.

In this study, we focused on three major bioactivities: antioxidant, anti-melanogenic, and anti-inflammatory effects. The antioxidant capacity of EVs was assessed both chemically and in cellular models. At the same time, anti-melanogenic activity was evaluated using α-MSH-stimulated melanoma cells, and anti-inflammatory effects were tested in keratinocytes, fibroblasts, and macrophages under stress or inflammatory stimuli. Finally, the in vivo relevance of *C. asiatica* EVs was investigated in a UVB-induced skin damage mouse model to assess their protective and restorative effects.

## 2. Results

### 2.1. Isolation and Characterization of C. asiatica EVs

**Figure 1:**
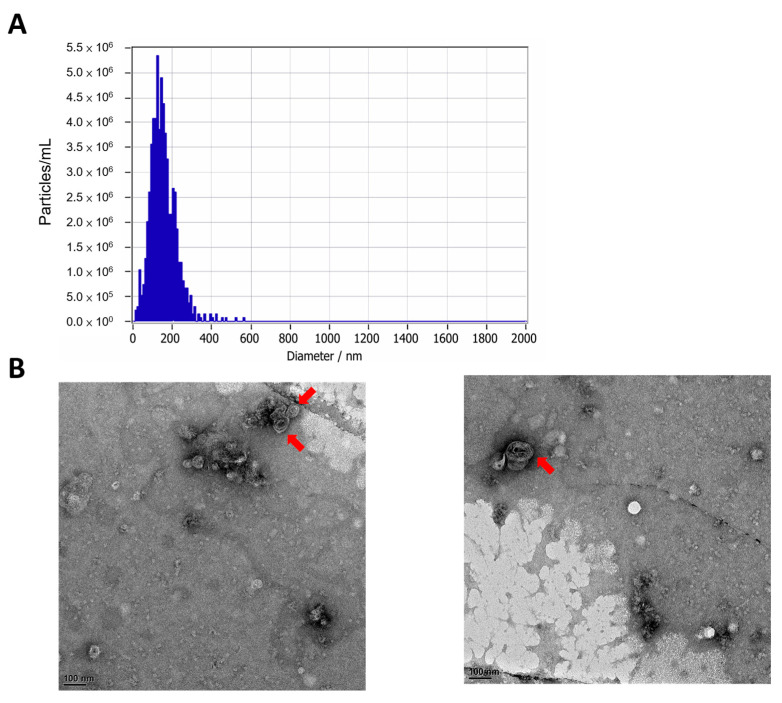
Size and morphology characterization of *C. asiatica*-derived EVs. (**A**) Nanoparticle tracking analysis (NTA) of size and concentration distribution of EVs by using the ZetaView^®^ system. (**B**) Representative TEM images of purified *C. asiatica* EVs with red arrows showing vesicles with lipid bilayer membrane structure. Scale bar: 100 nm.

*C. asiatica* EVs were isolated from tissue culture *C. asiatica* by centrifugation and filtration method. The size distribution of EVs harvested was analyzed by nanoparticle tracking analysis (NTA) and showed that the particle has a mean size of 150 nm (Figure 1A). The morphological features shown by the transmission electron microscopy (TEM) images can be observed as vesicles of approximately 100–150 nm in size with a lipid bilayer membrane structure (Figure 1B).

### 2.2. Cytotoxicity Assessment

**Figure 2:**
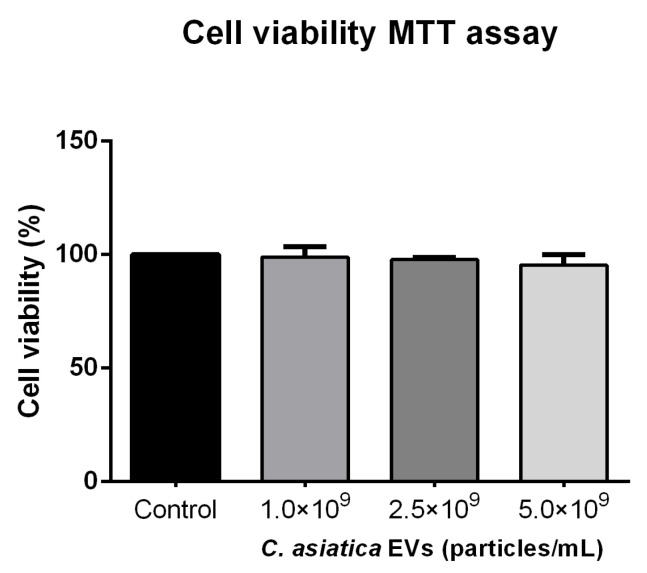
Cell viability assay. B16F10 cells were treated with different concentrations of *C. asiatica* EVs. After 24 h of treatment, cell viability was measured by the MTT assay method. Data are presented as mean ± SD (*n* = 3). Statistical results were calculated by one-way ANOVA with Dunnett’s multiple comparisons test compared to the control group.

To assess the possible cytotoxicity of *C. asiatica* EVs, B16F10 cells were treated with increasing concentrations of *C. asiatica* EVs and an MTT assay was performed. The results showed no significant differences compared to the control group in this test (Figure 2). Thus, high concentrations of up to 5 × 10^9^ particles/mL of *C. asiatica* EVs do not exhibit cytotoxicity in B16F10 cells. In addition, no discernible differences in cell viability were detected in HaCaT and Detroit 551 cell lines in the alamarBlue assay, confirming that the EVs do not exhibit cytotoxicity in mouse or human cells (Figure S1).

### 2.3. Total Polyphenol Content and Antioxidant Activity Assessment

#### 2.3.1. Total Polyphenol Content

The Folin–Ciocalteu assay was used to quantify the total polyphenol content of *C. asiatica* EVs as a composition parameter relevant to antioxidant potential. Mean polyphenol levels increased proportionally with EV concentration, ranging from 0.024 ± 0.001 to 0.091 ± 0.002 μg/mL gallic acid equivalents at 2 × 10^7^ to 5 × 10^7^ particles/mL, respectively (Figure S2). While total polyphenol content does not directly measure antioxidant activity, the results suggest that the phenolic constituents contained within EVs may contribute to their bioactivity.

#### 2.3.2. ABTS Radical Scavenging Assay

In the cell-free ABTS radical (ABTS^•+^) scavenging assay, *C. asiatica* EVs demonstrated dose-dependent antioxidant activity. At the highest EV concentration of 2 × 10^7^ particles/mL tested, EVs achieved 74.5% ± 0.39% scavenging activity, comparable to positive controls vitamin C (83.9% ± 0.26%) and even exceeded that of BHA (61.4% ± 0.04%) (Figure S3).

#### 2.3.3. Reducing Ability of Iron Chelating

In the potassium ferricyanide K_3_[Fe(CN)_6_] assay, *C. asiatica* EVs displayed potent dose-dependent metal chelating activity, reaching 84.8% ± 0.11% chelating activity at 1 × 10^7^ particles/mL compared to that of the positive control EDTA (98.7% ± 0.02%) (Figure S4).

#### 2.3.4. Intracellular Reactive Oxygen Species (ROS) Scavenging Assay

**Figure 3:**
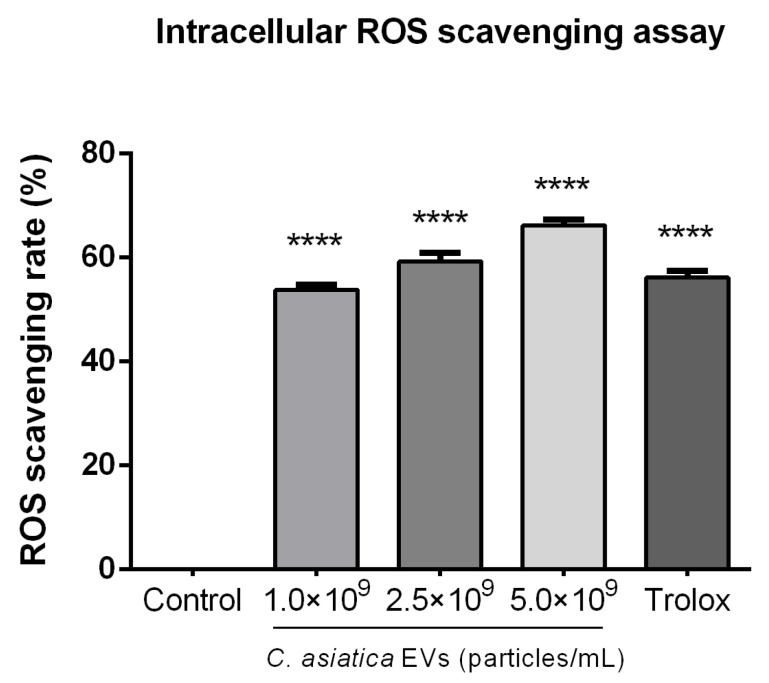
Intracellular ROS level. B16F10 cells were pretreated with various concentrations of *C. asiatica* EVs, Trolox (0.5 mg/mL, positive control), or left untreated as a control for 24 h. The cells were then incubated with 24 mM H_2_O_2_ and DCFH-DA. The fluorescence intensities of DCF were measured to calculate the ROS levels. Data are presented as mean ± SD (*n* = 3). Statistical analysis was calculated by one-way ANOVA with Dunnett’s multiple comparisons test, compared to the control group. **** *p* < 0.0001.

The antioxidant capacity of *C. asiatica* EVs was assessed in a cellular context in B16F10 cells subjected to hydrogen peroxide-induced oxidative stress. Pretreated cells with *C. asiatica* EVs significantly reduced intracellular ROS levels at 53.9% ± 0.94%, 59.3% ± 1.68% and 66.3% ± 1.10% for 1 × 10^9^, 2.5 × 10^9^ and 5 × 10^9^ particles/mL, respectively (Figure 3). Treatment by all concentrations of *C. asiatica* EVs was significantly more effective than the untreated control (*p* < 0.0001) and comparable to the Trolox control group (56.2% ± 1.35%).

### 2.4. Melanin Inhibitory Activity Assessment

#### 2.4.1. Melanin Content Assay

**Figure 4:**
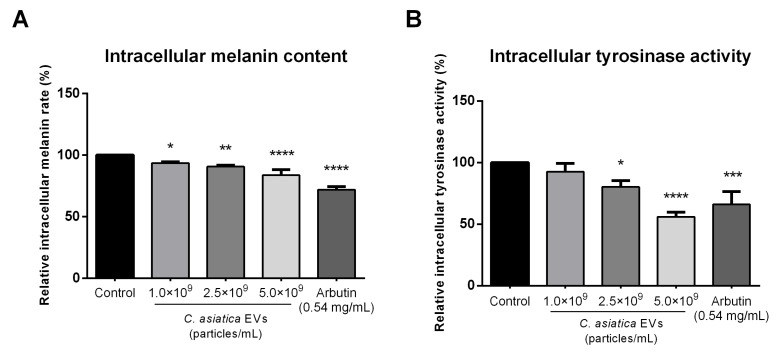
Effect of *C. asiatica* EVs on melanin production. Inhibitory effects of different concentrations of *C. asiatica* EVs on (**A**) melanin content and (**B**) intracellular tyrosinase activity in B16F10 cells. Data are presented as mean ± SD (*n* = 3). Statistical results were calculated by one-way ANOVA with Dunnett’s multiple comparisons test compared to the control group. * *p* < 0.05, ** *p* < 0.01, *** *p* < 0.001, **** *p* < 0.0001.

The ability of melanin synthesis inhibition by *C. asiatica* has been well documented [15]. Inhibition of melanin production was assayed in B16F10 melanoma cells treated with α-melanocyte-stimulating hormone (α-MSH). In α-MSH-stimulated B16F10 cells, *C. asiatica* EVs significantly reduced intracellular melanin content compared to untreated controls. Residual melanin levels were at 93.55% ± 1.14%, 90.73% ± 1.17% and 83.69% ± 4.31% for 1 × 10^9^, 2.5 × 10^9^ and 5 × 10^9^ particles/mL of EV, respectively (Figure 4A). On the other hand, positive control arbutin (0.54 mg/mL) reduced the melanin content to 71.95% ± 2.44%.

#### 2.4.2. Tyrosinase Activity Assay

As tyrosinase catalyzes the first two steps of melanin synthesis, we measured intracellular tyrosinase activity in B16F10 cells under identical conditions as in Section 2.4.1 [16].

*C. asiatica* EVs significantly inhibited tyrosinase, with activity levels at 92.5% ± 6.77%, 80.2% ± 5.13%, and 56.0% ± 3.83% at 1 × 10^9^, 2.5 × 10^9^ and 5 × 10^9^ particles/mL, respectively (Figure 4B). Notably, EVs at 5 × 10^9^ particles/mL were even more effective than arbutin (66.0% ± 10.53%) in suppressing tyrosinase activity, though they achieved less overall melanin reduction.

### 2.5. Effect of C. asiatica on Skin Integrity-Related Genes

#### 2.5.1. Type I Procollagen EIA

Type I procollagen (PIP) is a precursor of type I collagen secreted by skin fibroblasts, and lowered expression of PIP is associated with skin damage and senescence [17]. In Hs68 fibroblasts, *C. asiatica* EV treatment increased PIP production in a dose-dependent manner, with the highest concentration (1 × 10^9^ particles/mL) inducing levels comparable to the positive control, TGF-β1 (1634 ng/mL vs. 1642 ng/mL) (Figure S5).

#### 2.5.2. Aquaporin-3 and Filaggrin qRT-PCR

Aquaporin (AQP3) and filaggrin (FLG) are important in the barrier function of skin by regulating skin water retention [18]. qRT-PCR assay quantification of mRNA expression of AQP3 and FLG genes in HaCaT keratinocyte cells treated with *C. asiatica* EVs showed that EVs at 1 × 10^9^ particles/mL could upregulate the expression levels of AQP3 to 3.1-fold and FLG to 2.2-fold compared to the untreated control (Figure S6).

### 2.6. Anti-Inflammatory Effects of C. asiatica EVs

#### 2.6.1. COX2 Expression in UV-Irradiated Skin Cells

*C. asiatica* extract has previously been found to inhibit the expression of pro-inflammatory gene COX2 induced by UV [15]. Here, we showed that *C. asiatica* EV treatment reduced COX2 mRNA expression in UV-irradiated Hs68 skin fibroblasts and HaCaT keratinocytes compared to untreated UVB controls (Figure S7).

#### 2.6.2. Nitric Oxide Production in Macrophages

**Figure 5:**
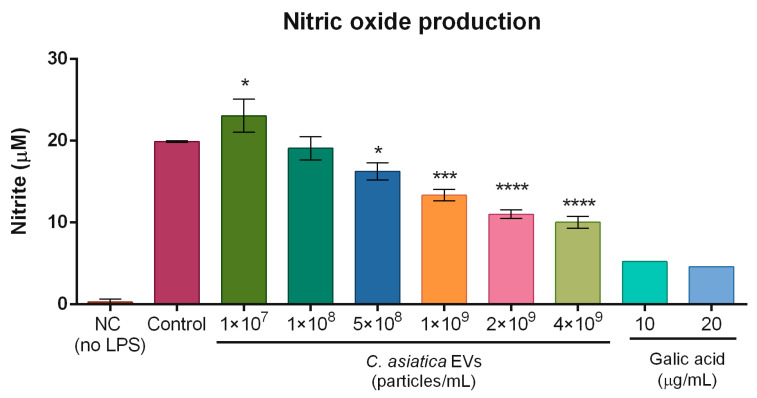
Nitric oxide (NO) production in RAW 264.7 macrophages. Cells were treated with different concentrations of *C. asiatica* EVs or gallic acid after induction by LPS. The resulting NO produced was detected by the Griess reagent. Data are presented as mean ± SD (*n* = 3). Statistical results were calculated by one-way ANOVA with Dunnett’s multiple comparisons test compared to the control group. * *p* < 0.05, *** *p* < 0.001, **** *p* < 0.0001.

Nitric oxide (NO) is an inflammatory response messenger elevated in response to skin UV irradiation [19]. Lipopolysaccharide (LPS) can stimulate NO production in macrophages [20]. In LPS-stimulated RAW 264.7 macrophages, *C. asiatica* EV treatment significantly reduced nitrite levels (as a proxy for NO production) in a dose-dependent manner (Figure 5). The highest concentration tested (4 × 10^9^ particles/mL) resulted in a marked decrease compared to the LPS-only control (10.04 ± 0.74 µM vs. 19.90 ± 0.12 µM, *p* < 0.0001).

### 2.7. Effect of C. asiatica EV-Based Gel on UVB-Induced Skin Damage in MICE

#### 2.7.1. Safety and Tolerability

First, we evaluated the safety of the application of a *C. asiatica* EV gel applied onto the back of mice after UVB irradiation. All *C. asiatica* EV-based gel formulations were well tolerated, with no signs of skin irritation or adverse effects in any treatment group, regardless of the addition of EV or TECA (Table S1). There were also no significant weight differences between groups (*p* > 0.05), despite a slight decrease in body weight observed in all groups during the study (Table S2 and Figure S8).

#### 2.7.2. Epidermal Thickness

**Figure 6:**
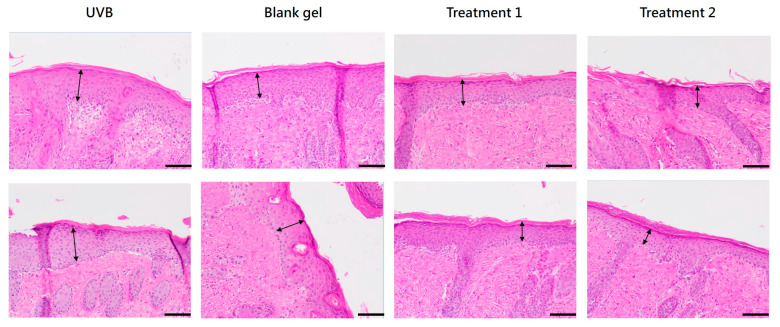
Representative examples of histopathological analysis of skin tissue sections on Day 7 of the study. Arrows indicate the thickness of the epidermis. H&E stain, 200× magnification (scale bar = 100 µm).

Histological analysis showed that *C. asiatica* EV and EV + TECA gels reduced epidermal thickness to 87.66 ± 35.32 μm and 86.09 ± 25.74 μm, respectively, compared to blank gel control (154.94 ± 58.14 μm) (Table 1, Figure 6). The epidermal thickness of treatment groups was also lower than that of the UVB-only group (126.66 ± 47.73 μm).

#### 2.7.3. Inflammatory Cell Infiltration

**Figure 7:**
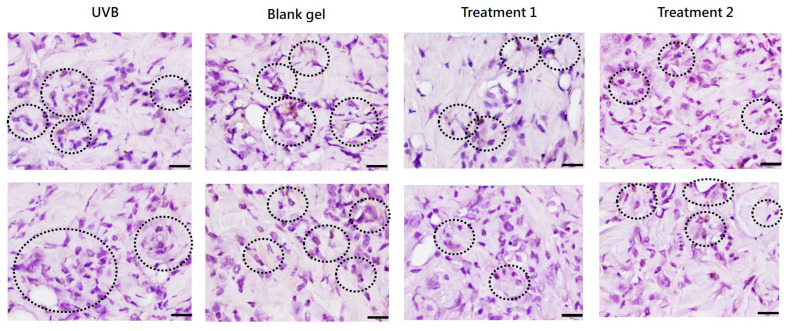
Representative examples of IHC staining of myeloperoxidase (in brown, indicated by circles) in skin tissue sections on Day 7 of the study. Mouse back skin sections were stained with rabbit anti-mouse myeloperoxidase antibody and then detected by HRP goat anti-rabbit antibody. 400× magnification (scale bar = 20 µm).

In addition, immunohistochemical (IHC) staining of inflammation marker myeloperoxidase (MPO) was used to observe positive multinucleated cells in the dermis of UV-damaged areas. MPO staining revealed substantial infiltration in 75% of the fields for UVB-only and blank gel groups, versus only 8.3% for the EV or EV + TECA-treated groups (Table 1 and Figure 7).

#### 2.7.4. Gross Skin Appearance

**Figure 8:**
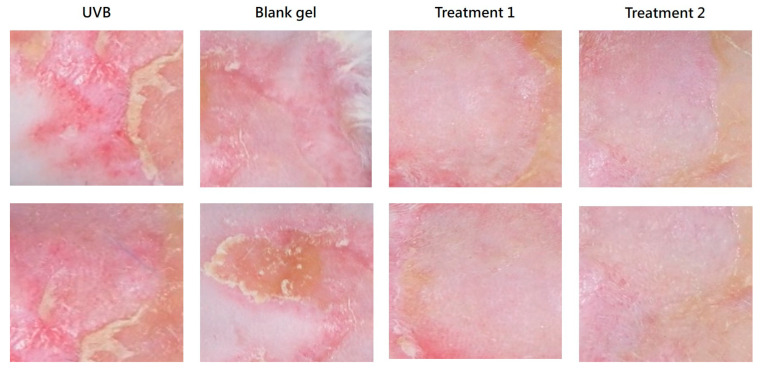
Representative examples of mouse skin appearance at the site of UVB exposure on Day 6 of the study.

On Day 6, the EV and EV + TECA gel-treated groups showed improved wound closure with minimal scarring and the absence of inflammation. In contrast, UVB-only and blank gel groups showed persistent redness with only a few newly repaired skin and abundant scarring (Figure 8).

## 3. Discussion

In this study, we successfully isolated and characterized EVs from tissue-cultured *C. asiatica* and evaluated their bioactivity using in vitro assays and an in vivo UVB-induced skin damage model. The EVs were of typical lipid bilayer vesicle morphology with an average diameter of approximately 150 nm, consistent with previously reported PDEVs [12,13]. Notably, the EVs were non-cytotoxic to multiple skin cell lines, including HaCaT keratinocytes, Detroit 551 fibroblasts, and B16F10 melanocytes (Figure 2 and Figure S1), in contrast to previously reported smaller *C. asiatica* EVs (~63.8 nm), which showed cytotoxicity in HepG2 hepatocellular carcinoma cells [21]. This is also in agreement with other studies showing that plant EVs from sources such as *Dendropanax morbifera* and grape are generally well tolerated by mammalian cells [9,22].

Traditionally, the bioactivity of *C. asiatica* is attributed to triterpenes such as madecassoside and asiaticoside in the plant extract. At the same time, our findings suggest that the encapsulated content within EVs (including proteins, phytochemicals, and RNAs) may offer additional or synergistic effects to phytochemicals [3,4,5]. Although this study did not profile miRNA and other small nucleic acids, EV-associated nucleic acids are important modulators of gene expression, which are packaged by RNA-binding proteins [10,22].

We observed dose-dependent antioxidant activities in *C. asiatica* EVs in cell-free (ABTS and metal chelation) and intracellular (ROS reduction) assays (Figure 3, Figure S3 and Figure S4). The magnitude of ABTS radical scavenging (74.5% at 2 × 10^7^ particles/mL) was comparable to that of vitamin C. It exceeded BHA, in line with prior work on *D. morbifera* EVs, which also showed strong radical scavenging capacity [9]. Our observation that EV polyphenol content increased with particle concentration (Figure S2) supports earlier findings that phenolic compounds in plant EVs contribute to antioxidant properties [8,9].

In terms of anti-inflammatory action, *C. asiatica* EVs reduced UVB-induced COX2 expression in fibroblasts and keratinocytes, and lowered LPS-induced NO production in macrophages (Figure 5 and Figure S7). These results parallel reports that *C. asiatica* extract suppressed COX2 expression in UV-irradiated keratinocytes, and plant EVs from ginseng and ginger were also able to reduce NO production in RAW 264.7 cells [8,11,15]. Our data extend these observations by showing comparable activity of *C. asiatica* EVs across multiple skin cell types.

Previous studies demonstrated that madecassoside from C. asiatica extracts inhibited UV-induced melanogenesis in keratinocyte and melanocyte co-cultures and human skin [15]. We found that *C. asiatica* EVs suppressed α-MSH–induced melanin production and intracellular tyrosinase activity in B16F10 cells (Figure 4). While melanin reduction was less than that achieved by arbutin, tyrosinase inhibition was similar or greater at higher EV concentrations. These results are consistent with other plant EVs, which also reduced melanin synthesis via tyrosinase pathway suppression [9,22]. As inflammation can contribute to pigment production, suppression of COX2 and NO suggests an anti-inflammatory mechanism to melanin reduction [23,24]. *C. asiatica* EVs upregulated expression of PIP1, AQP3, and FLG (Figures S5 and S6). These genes are essential for collagen synthesis and skin barrier function, and their upregulation is consistent with a transcriptomic study on *C. asiatica* EV that identified upregulation of AQP3, FLG, and other barrier-related genes in human keratinocytes, along with 11 novel miRNAs likely contributing to these effects [13].

Photoaging leads to degeneration of elastic fibers and collagen loss, resulting in epidermal thickening of the stratum corneum [23]. Daily topical application of *C. asiatica* EV and EV + TECA gels for 7 days reduced UVB-induced epidermal thickening and MPO-positive inflammatory cell infiltration compared to both UVB-only and blank gel controls (Figure 6 and Figure 7, Table 1). These effects conform with prior reports where *C. asiatica* extract reduced histological damage and inflammatory markers in UVB-exposed mouse skin [15]. The slight increase in epidermal thickness in the blank gel group compared to UVB-only controls may reflect mild mechanical irritation from gel application. Although our EV gel treatment did not fully reverse epidermal changes within 7 days, the mitigation of UVB damage and increased recovery pace are promising for longer-term studies.

These preclinical data are supported by our preliminary clinical data from a 28-day pilot trial, where a topical *C. asiatica* EV–based serum formulation significantly improved hydration, elasticity, pigmentation, redness, and pore size [24]. These clinical results mirrored our observations in the preclinical study, such as upregulated AQP3, FLG, and PIP genes correlating with the increased hydration and elasticity in clinical observations. The significant decreases in melanin content and skin redness in trial participants also complement the in vitro findings of melanin synthesis suppression and anti-inflammatory activities. Notably, the tissue culture method used to produce *C. asiatica* EVs offers a scalable, genetically uniform, and environmentally sustainable alternative to wild harvesting [14,25], consistent with best practices for reproducibility and ecological responsibility. Moreover, tissue culture systems can be optimized to boost yields of specific bioactives using elicitors [14].

This study has several limitations. First, although we demonstrated the biological activity of *C. asiatica* EVs in vitro and in vivo, the precise molecular mechanisms, such as the role of small RNAs or miRNAs within the EVs, remain to be defined. While we confirmed the morphology and size distribution of *C. asiatica* EVs using TEM and NTA, we did not perform marker analysis or negative controls in accordance with MISEV2023 recommendations [26]. Protein-to-particle ratios and canonical EV marker assays (e.g., immunoblotting) would help further validate EV purity, although we acknowledge that standardized markers for plant-derived EVs have yet to be defined, unlike mammalian tetraspanins CD9 and CD63 [8,9]. Second, the skin penetration and stability of the EVs in the topical gel formulation have not been evaluated. Third, the animal study involved a relatively small sample size (*n* = 3 per group); thus, it was not powered or robust for statistical analysis, and we were only able to present data descriptively. The limited observation period of 7 days may also not fully capture the long-term and broader effects of EVs on skin quality and inflammatory responses. Furthermore, the absence of a non-UVB–exposed control group in the animal study limited our ability to assess baseline inflammation and epidermal integrity. This non-UVB group baseline would have improved the comparison of histopathological changes and epidermal recovery. Thus, while the animal results support a trend toward efficacy, they should be regarded as preliminary at this point. Finally, although antioxidant and NO assays were performed with appropriate blanks, the contents of EVs could chemically interfere with radical scavenging or Griess reactions. Future research should include transcriptomic or proteomic profiling of EV content and compare it to that of a previous study on *C. asiatica* EV transcriptome analysis. Longer-term and larger-scale in vivo studies, with non-UVB control groups included, will also require investigation.

## 4. Materials and Methods

### 4.1. Isolation and Characterization of Centella asiatica EVs

EVs were isolated from tissue-cultured *C. asiatica* by a multi-step filtration and centrifugation method. Briefly, fresh tissue culture *C. asiatica* was homogenized, coarsely filtered to remove large debris, and centrifuged. Pellets were collected, filtered through a 0.45 μm filter, extracted and finally filtered through a 0.45/0.22 μm membrane filter.

The size and morphology of the *C. asiatica* EVs were analyzed with nanoparticle tracking analysis (NTA) and transmission electron microscopy (TEM). The NTA was performed with the ZetaView^®^ system (Particle Metrix, Inning am Ammersee, Germany). For TEM, 10 µL of EV suspension was applied to carbon-coated copper grids, allowed to adsorb for 2 min, and blotted dry with filter paper. Negative staining was performed twice with 10 µL of 2% uranyl acetate, each followed by blotting. Grids were air-dried and imaged under TEM.

### 4.2. Cell Culture

The following cell lines were used in the study:

- B16F10 (mouse melanoma, BCRC #60031, ATCC CRL6475) was maintained in Dulbecco’s Modified Eagle Medium (DMEM) (Hyclone, Cytiva, Marlborough, MA, USA) supplemented with 10% fetal bovine serum (FBS) and 1% antibiotics (Penicillin and streptomycin, Pen-Strep, Cat. #15070063, Gibco, Thermo Fisher Scientific, Vantaa, Finland).
- HaCaT (human keratinocyte, AddexBio Cat. #T0020001, San Diego, CA, USA) was grown in high-glucose DMEM supplemented with 10% FBS, 4 mM/L-glutamine, and 1% antibiotic-antimycotic solution (Cat. #15240062, Gibco, Thermo Fisher Scientific).
- Hs68 (human foreskin fibroblast, BCRC #60038) was grown in the same conditions as HaCaT
- Detroit 551 (human fibroblasts, BCRC #60118) was grown in Minimum Essential Medium (MEM) (Hyclone, Cytiva, Marlborough, MA, USA) supplemented with 10% FBS, 0.1 mM non-essential amino acids, and 1.0 mM sodium pyruvate.
- RAW 264.7 (mouse macrophage, BCRC #60001) was maintained in DMEM containing 10% FBS and 1% antibiotics (Pen-Strep Cat. #15070063, Gibco, Thermo Fisher Scientific).

All cells were maintained at 37 °C in a humidified atmosphere containing 5% CO_2_. Cell lines (except for HaCaT, which was purchased from AddexBio, San Diego, CA, USA) were obtained from the Bioresource Collection and Research Center (BCRC), Hsinchu City, Taiwan.

### 4.3. Cell Viability Assay

MTT assay (B16F10): Cells were seeded into 24-well plates at a density of 5 × 10^4^ cells per well and incubated for 24 h. After reaching ~70% confluence, the cells were treated with *C. asiatica* EVs at various concentrations for 24 h. After treatment, the culture medium was removed and cells were washed twice with PBS, and 500 μL of MTT solution (0.5 mg/mL in PBS) (Sigma-Aldrich Cat. #475989, Merck KGaA, Darmstadt, Germany) was added and incubated for 4 h at 37 °C. The insoluble crystals were then solubilized with 500 μL of ethanol-DMSO (1:1) mixture and shaken for 30 s. The absorbance of the wells at 570 nm was read using a Multiskan GO microplate reader (Thermo Fisher Scientific).

alamarBlue (resazurin)assay (HaCaT, Detroit 551): After 24 h of the EV treatment, 1/10 volume of alamarBlue^®^ (Cat. #DAL1100, Thermo Fisher Scientific) was added to the culture medium and incubated for 4 h at 37 °C. The absorbance was then read at 570 nm with 600 nm as a reference using a microplate reader.

In both assays, cell viability was expressed as the ratio of the absorbance of the treated cells to that of the controls.

### 4.4. Thiazoline-6-Sulfonic Acid (ABTS) Free Radical Scavenging Assay

Antioxidant activity was measured using the ABTS free radical assay [27]. Briefly, ABTS radical cation (ABTS^•+^) was generated by reacting 7 mM ABTS stock solution (Sigma-Aldrich Cat. #A1888) with 2.45 mM potassium persulfate (Sigma-Aldrich Cat. #216224) and incubated in the dark at room temperature for at least 6 h before use. For the assay, 1 mL of the above ABTS^•+^ solution was mixed with *C. asiatica* EVs at 4 × 10^6^, 1 × 10^7^, and 2 × 10^7^ particles/mL concentrations, and the absorbance at 734 nm was measured immediately after mixing within 10 min using BioTek Epoch spectrophotometer (BioTek, Agilent Technologies, Winooski, VT, USA). Vitamin C (ascorbic acid, 0.9 mg/mL, Sigma-Aldrich Cat. #A7506) and BHA (butylated hydroxyanisole, 0.9 mg/mL, Sigma-Aldrich Cat. #B1253) were used as positive controls.

The result was presented as a percentage of ABTS^•+^ radical scavenging activity calculated using the following formula:

$$Scavenging\;activity\;\left(\%\right)=\left(1-\frac{A_{sample}}{A_{control}}\right)\times 100$$

*A_control_* is the absorbance of the ABTS^•+^ solution without sample, and *A_sample_* is the absorbance after adding *C. asiatica* EVs or controls.

### 4.5. Intracellular Reactive Oxygen Species (ROS) Assay

Intracellular ROS levels were measured using the 2′, 7′-dichlorofluorescein diacetate (DCFH-DA) method [28]. B16F10 cells were seeded in 24-well plates at a density of 0.5 × 10^5^ cells/well in DMEM with 10% FBS under standard culturing conditions. Twenty-four hours after seeding, the cells were treated for 24 h with *C. asiatica* EVs at concentrations of 1 × 10^9^, 2.5 × 10^9^, and 5 × 10^9^ particles/mL, Trolox® (0.5 mg/mL, positive control, Sigma-Aldrich Cat. #648471), or untreated (negative control). After treatment, the culturing medium was aspirated, and the cells were washed twice with PBS and incubated with 20 mM H_2_O_2_ for 30 min at 37 °C to induce oxidative stress. After incubation, cells were detached with trypsin/EDTA (Hyclone, Cytiva, Marlborough, MA, USA), pelleted by centrifugation, and resuspended in 10 µM DCFH-DA solution (Sigma-Aldrich Cat. #D6883) and incubated for 30 min at 37 °C in the dark. Following incubation, the reaction mixtures were transferred into a black 96-well plate, and fluorescence intensity was measured using a Fluoroskan Ascent fluorescent reader (Thermo Fisher Scientific) with excitation at 485 nm and emission at 538 nm. ROS scavenging rate was expressed as a percentage of the untreated (negative) control group.

### 4.6. Total Polyphenol Content Assay

Total polyphenol content was determined using the Folin–Ciocalteu method using gallic acid standard [29]. A gallic acid standard curve was prepared by dissolving gallic acid (Sigma-Aldrich, Cat. #G7384) in deionized water with concentrations ranging from 0 to 100 μg/mL. *C. asiatica* EVs at 2 × 10^7^, 4 × 10^7^, 5 × 10^7^ particles/mL and gallic acid standards were mixed with Folin–Ciocalteu reagent (Sigma-Aldrich Cat. #1.09001) and incubated for 5 min. After incubation, 7% (*w*/*v*) sodium carbonate solution (Sigma-Aldrich Cat. #S8761) was added and incubated for 90 min in the dark. The absorbance at 765 nm was measured using a BioTek Epoch spectrophotometer (BioTek), and the total polyphenol content was expressed as μg/mL gallic acid equivalent using the standard curve.

### 4.7. Metal Chelating Assay

The metal chelating activity of the extracellular vesicles was assessed with the potassium ferricyanide (K_3_[Fe(CN)_6_]) reducing assay [30]. *C. asiatica* EVs at 2 × 10^6^, 5 × 10^6^, 1 × 10^7^ particles/mL were mixed with 0.2 mM PBS and 1% (*w*/*v*) potassium ferricyanide (Fluka Cat. #702587, Honeywell, Charlotte, NC, USA). EDTA (2 mg/mL, J.T.Baker Cat. #JT8993-1, Avantor Inc., Radnor, PA, USA) was used as the positive control, and deionized water was used as the blank control. The mixtures were heated at 50 °C for 20 min, cooled to room temperature, then mixed with 10% (*w*/*v*) trichloroacetic acid (TCA, Sigma-Aldrich Cat. #T6399), and centrifuged (12,000 rpm, 4 °C for 5 min). The supernatants were transferred to a 96-well plate, mixed with deionized water and 1% (*w*/*v*) FeCl_3_, and incubated in the dark for 30 min. Absorbance was measured at OD 700 nm using a BioTek Epoch spectrophotometer (BioTek) to assess metal chelating activity.

The result was presented as a percentage of chelating activity relative to EDTA (as 100%).

### 4.8. Intracellular Melanin Content Assay

The intracellular melanin content was measured as described previously [31]. B16F10 cells were seeded into 24-well plates at a density of 5 × 10^4^ cells per well in culture medium supplemented with 100 nM α-Melanocyte-stimulating hormone (α-MSH, Sigma-Aldrich Cat. #M4135) for 24 h to induce melanin production. On the following day, the cells were treated for an additional 24 h with *C. asiatica* EVs (1 × 10^9^, 2.5 × 10^9^, and 5 × 10^9^ particles/mL), or 0.54 mg/mL arbutin (Sigma-Aldrich Cat. #A4256) as a positive control. After treatment, the culture medium was removed and the cells were washed twice with PBS. The cells were then solubilized in 1 N NaOH containing 10% DMSO at 60 °C for 60 min in the dark. The lysates were then transferred to a 96-well plate, and melanin content was assayed at 405 nm using a BioTek Epoch spectrophotometer (BioTek). Melanin levels were expressed relative to untreated controls.

### 4.9. Intracellular Tyrosinase Activity Assay

The intracellular tyrosinase activity was determined as described previously [32]. B16F10 cells were cultured and stimulated with α-MSHm, then treated with *C. asiatica* EVs or controls in identical conditions to the above melanin content assay (Section 4.8). After treatment, the culturing medium was removed, and the cells were washed twice with PBS. The cells were lysed with lysis buffer (50 mM sodium phosphate buffer, 1 mM PMSF, 1% Triton X-100) and subjected to 2–3 freeze–thaw cycles at −80 °C to ensure thorough cell disruption. Lysates were then centrifuged at 12,000 rpm for 20 min at 4 °C, and the supernatant was transferred to a 96-well plate and mixed with freshly prepared L-DOPA solution (1mg/mL L-DOPA in PBS, Sigma-Aldrich Cat. #D9628) and incubated at 37 °C for 60 min in the dark. The absorbance at 490 nm was then measured using a BioTek Epoch spectrophotometer (BioTek) to assess the tyrosinase activity. Results were expressed as relative tyrosinase activity compared to the untreated control.

### 4.10. Type I Procollagen (PIP) ELISA Assay

Hs68 cells were seeded at 1 × 10^5^ cells per well in 12-well plates and cultured in 10% FBS DMEM at 37 °C with 5% CO_2_ for 24 h. After three washes with PBS, 1 × 10^7^, 1 × 10^8^, 1 × 10^9^ particles/mL of *C. asiatica* EVs or 5 ng/mL TGF-β1 (positive control, AbCam Cat. #ab50036, Cambridge, UK) were added and cultured for 24 h. The level of type I procollagen in cell culture supernatants (in ng/mL) was determined using a commercial PIP EIA Kit (TAKARA Cat. # MK101, Kyoto, Japan) according to the manufacturer’s instructions (2-step procedure) using a Multiskan GO microplate reader (Thermo Fisher Scientific).

### 4.11. Gene Expression of Aquaporin-3 (AQP3) and Filaggrin (FLG)

HaCaT cells were seeded into a 96-well plate and cultured for 24 h. Cells were treated with various concentrations of *C. asiatica* EVs or purified water (negative control) for 24 h. After treatment, culture medium was removed and cells were washed once with PBS and lysed with SuperPrep TM cell lysis & RT Kit for qPCR (Cat. #SCQ-101, TOYOBO, Tokyo, Japan). Eight μL of the above-extracted lysate was added to 32 μL of the RT-PCR reaction mixture, and cDNA was synthesized under conditions of 37 °C for 15 min, 50 °C for 5 min, and 95 °C for 5 min using the above SuperPrep TM cell lysis & RT Kit for qPCR (TOYOBO). Gene expression levels were analyzed with quantitative PCR (qPCR) using Thunderbird TM SYBR qPCR Mix (Cat. #QPS-201, TOYOBO) with Qiagen QuantiTect primer (AQP3, Cat. #QT00212996; FLG, Cat. #QT02448138; GAPDH, Cat. #QT01192646, QIAGEN, Venlo, Limburg, The Netherlands). qPCR cycling conditions were: 95 °C for 1 min, followed by 40 cycles of 94 °C for 15 s, 60 °C for 30 s, and 72 °C for 30 s using QuantStudio 5 thermocycler (Thermo Fisher Scientific). The gene expression levels of the sample were quantified by normalization with GAPDH using the 2^−∆∆Ct^ method.

### 4.12. mRNA Expression of the COX2 Gene After UVB Induction

HaCaT cells (1 × 10^4^/well for 96-well) or Hs68 cells (5 × 10^4^/well for 24-well) were cultured for 24 h and exposed to UVB at a 90 mJ dose using BLX-312 UVB irradiation system (Vilber, Marne-la-Vallée, France). After UVB exposure, *C. asiatica* EVs and negative control (purified water only) were added to the cells and incubated for 4 h. qRT-PCR was performed as in 4.11 above to quantify the expression of cyclooxygenase-2 (COX2) gene using Qiagen’s QuantiTect primer for COX2 (Cat. #QT00040586, QIAGEN). The gene expression levels were quantified by normalization with GAPDH using the 2^−∆∆Ct^ method.

### 4.13. Measurement of Nitric Oxide Production

RAW 264.7 macrophage cells were seeded in 10% FBS DMEM in 24-well plates at a density of 2 × 10^5^ cells/well and cultured for 24 h. After removing the culture medium, the cells were treated with 1 × 10^7^, 1 × 10^8^, 5 × 10^8^, 1 × 10^9^, 2 × 10^9^, or 4 × 10^9^ particles/mL of *C. asiatica* EVs or gallic acid (10 μg/mL and 20 μg/mL, Sigma-Aldrich Cat. #G7384) in serum-free DMEM and incubated for 4 h. After pre-incubation, the cells were stimulated with 100 ng/mL LPS (Sigma-Aldrich Cat. #L3129) and incubated for 20 h. The culture supernatants were harvested and centrifuged at 7000× *g* for 3 min. The samples were mixed with an equal volume (100 μL) of Griess reagent (Sigma-Aldrich Cat. #G4410), and the absorbance was measured at 540 nm after reacting for 10 min using a Multiskan GO microplate reader (Thermo Fisher Scientific).

### 4.14. Animal Study

Twelve 7-week-old (31 to 33 g weight) male ICR mice were purchased from BioLASCO Taiwan Co., Ltd. (Taipei City, Taiwan) and housed in a GLP-compliant animal research facility under controlled conditions (temperature: 22 ± 4 °C; humidity: 30–70%; 12 h light/dark cycle). Mice had unlimited access to autoclaved water and standard laboratory chow, LabDiet 5058 PicoLab^®^ Mouse Diet 20 (Land O’Lakes Inc., Arden Hills, MN, USA). After 5 days of acclimatization, the mice were randomly assigned to four groups as shown in Table 2.

On Day 0, mice were anesthetized with isoflurane (3–4%), and back hair was shaved and further removed using hair removal cream. UVB-induced skin damage was performed by exposing mice to 120 mJ/cm^2^ UVB for 5 min using a UVP CL-1000M UV Crosslinker (Analytik Jena GmbH+Co. KG, Jena, Germany). Immediately after UVB exposure, 180–200 μL of test gel was applied twice daily topically on the back according to the respective group assignments.

Photographs of the dorsal skin were taken before UVB exposure on Day 0 and daily from Day 1 to Day 7 prior to gel application. The body weight of the animals was measured on Days 0, 1, 4, and 7. Animals were sacrificed on Day 7 for histopathological analysis.

The composition of the base gel was as follows: Carbomer 934, polyethylene glycol (PEG) 400, glycerol, triethanolamine, phenoxyethanol, and 0.9% (*w*/*v*) sodium chloride solution. *C. asiatica* EVs were produced as outlined in 4.1, and TECA was obtained from SEPPIC (La Garenne-Colombes, France).

### 4.15. Animal Tissue Histopathological Analysis

Dorsal skin tissue samples were collected at sacrifice, fixed in 10% neutral formalin for at least 24 h, paraffin-embedded, and sectioned. After gradient alcohol dehydration, xylene permeation, and paraffin embedding, the skin tissue was cut into several continuous thin sections, each 4–6 µm thick. The long axis of the skin tissue section was defined as the observation direction. The first set of slides was stained with H&E, and five random fields were photographed at 200× magnification. Epidermal thickness was measured six times per field. Immunohistochemistry (IHC) staining was performed on the second set of sections, which were rehydrated, and antigen retrieval was performed using a pH 6 recovery solution. Slides were then incubated with rabbit anti-mouse myeloperoxidase antibody (Ab65871, Abcam Ltd., Cambridge, UK) at a concentration of 1 µg/mL, followed by goat anti-rabbit secondary antibody (GTX83399, GeneTEX, Taipei, Taiwan) to stain antigen-positive cells in the dermis with hematoxylin stain as a counterstain. Six dermal areas were observed at 400× magnification.

Infiltrating cell counts were categorized into five grades according to ISO 10993-6:2016: Table E1 [33]:

- 0 points (negative)
- 1 (1–5 positive cells per field)
- 2 (6–10 positive cells per field)
- 3 (abundant infiltration of positive cells)
- 4 (field densely packed with positive cells)

All histological measurements and assessments were performed by a histologist blinded to the grouping of animals.

All animal works were supervised by a licensed veterinarian and performed in a GLP-compliant animal testing facility. All procedures involving study animals were conducted in a manner that avoided or minimized discomfort, distress, or pain to the animals. Clinical symptoms were observed once per day on behavior, respiration, and appearance. The animal study protocol (Approval No. 113071) was approved on 24 October 2024 by the Institutional Animal Care and Use Committee (IACUC) of Agricultural Technology Research Institute (Taipei City, Taiwan).

### 4.16. Statistical Analysis

Data are presented as mean values ± SD. The analysis package in GraphPad Prism 6.01 (Boston, MA, USA) was used for statistical analysis. Statistical results were calculated by one-way ANOVA with Dunnett’s multiple comparisons test.

## 5. Conclusions

This study demonstrated the antioxidant, anti-melanogenic and anti-inflammatory potential of EVs derived from *C. asiatica* in tissue culture. Compared to conventional botanical extracts, EVs derived from tissue culture offer a sustainable, reproducible and potentially more effective alternative with enhanced cellular delivery capabilities. Future research should focus on characterizing the key bioactive molecules and miRNAs within EVs responsible for these effects. Further investigation of the long-term safety, efficacy, and skin penetration of EV-based formulations in both animal models and clinical studies is also warranted. Ultimately, this work paves the way for the sustainable production and utilization of PDEVs in skin care cosmetics and wound-healing applications.

## Figures and Tables

**Table 1:** Tissue section measurement and observation. Results are taken from measurements of two representative skin sections (5 random fields for H&E stain, 6 random fields for IHC stain).

	UVB	Blank Gel	Treatment 1	Treatment 2
H&E stain-epidermis thickness (mean ± SD in µm)				
Results	126.66 ± 47.73	154.94 ± 58.14	87.66 ± 35.32	86.09 ± 25.74
IHC stain-Number of positives/Total number observed				
Myeloperoxidase-positive (score ≥ 3)	9/12	9/12	1/12	1/12

**Table 2:** Group assignment for the animal study.

Groups	Description	#Number of Mice
UVB	UVB exposure only	3
Blank gel	UVB exposure + base gel (without active ingredients)	3
Treatment 1	UVB exposure + base gel plus purified *C. asiatica* EVs (1 × 10^9^ particles/mL)	3
Treatment 2	UVB exposure + base gel plus purified *C. asiatica* EVs (1 × 10^9^ particles/mL) and TECA (Titrated Extract of *C. asiatica*)	3

## Data Availability

Data is provided within the manuscript and Supplementary Information.

## Supplementary Materials

The following supporting information can be downloaded at: https://www.mdpi.com/article/10.3390/ijms26188982/s1.
