# Supplementary material for: In Vitro Characterization of Centella asiatica Extracellular Vesicles and Their Skin Repair Effects in a UVB-Irradiated Mouse Model
Source: Int J Mol Sci. 2025 Sep 15;26(18):8982. doi: 10.3390/ijms26188982 (PMC12469282; doi:10.3390/ijms26188982)
Supplement: Supplementary file 1 [file ijms-26-08982-s001.zip › ijms-3759202-supplementary/Supplemental Tables v2.1.pdf]

Table S1 Clinical observation of animals [(1)]

Groups	Animals exhibiting abnormality							Total occurrence rate (N/N) [(2)]
	Day 1	Day 2	Day 3	Day 4	Day 5	Day 6	Day 7	
UVB	0	0	0	0	0	0	0	0/3
Blank gel	0	0	0	0	0	0	0	0/3
Treatment 1	0	0	0	0	0	0	0	0/3
Treatment 2	0	0	0	0	0	0	0	0/3

[(1)]Clinical observation items： Appearance, mood, behavior, respiration, mouth and nose, eyes, skin, digestion, or metabolism

[(2)] N/N： Number of abnormal animals/Total number of animals observed

Table S2 Body weight of animals over the course of the study (in grams).

**Groups**	**Animal**	**Day 0**	**Day 1**	**Day 4**	**Day 7**
**UVB**	B1	36.51	38.56	33.82	33.15
	B2	33.62	35.22	31.85	32.91
	B3	32.50	35.04	32.36	32.68
	**Mean**	**34.21**	**36.27**	**32.68**	**32.91**
	**SD**	**2.07**	**1.98**	**1.02**	**0.23**
**Blank gel**	R1	33.88	34.44	34.08	33.55
	O2	33.12	33.64	32.57	31.82
	R3	36.37	37.48	36.18	34.65
	**Mean**	**34.46**	**35.19**	**34.28**	**33.34**
	**SD**	**1.70**	**2.03**	**1.81**	**1.43**
**Treatment 1**	**O1**	34.93	35.84	35.57	34.54
	**O3**	32.52	33.31	31.63	31.76
	**O4**	35.22	36.90	36.03	34.85
	**Mean**	**34.22**	**35.35**	**34.41**	**33.72**
	**SD**	**1.48**	**1.84**	**2.42**	**1.70**
**Treatment 2**	**G1**	35.90	36.51	35.18	35.09
	**G2**	34.22	35.32	33.77	33.73
	**G4**	32.56	32.51	31.31	31.85
	**Mean**	**34.22**	**34.78**	**33.42**	**33.56**
	**SD**	**1.67**	**2.05**	**1.96**	**1.63**
